# Quantitative trait locus mapping and candidate gene analysis for salt tolerance at bud stage in rice

**DOI:** 10.3389/fpls.2022.1041081

**Published:** 2023-01-16

**Authors:** Wenjing Yin, Tianqi Lu, Zhengai Chen, Tao Lu, Hanfei Ye, Yijian Mao, Yiting Luo, Mei Lu, Xudong Zhu, Xi Yuan, Yuchun Rao, Yuexing Wang

**Affiliations:** ^1^ College of Chemistry and Life Sciences, Zhejiang Normal University, Jinhua, Zhejiang, China; ^2^ State Key Laboratory of Rice Biology, China National Rice Research Institute, Hangzhou, Zhejiang, China

**Keywords:** rice, germination rate, salt stress, QTL mapping, recombinant inbred line population

## Abstract

Soil salinization has a serious influence on rice yield and quality. How to enhance salt tolerance in rice is a topical issue. In this study, 120 recombinant inbred line populations were generated through nonstop multi-generation selfing using a male indica rice variety Huazhan (*Oryza sativa* L. subsp. *indica* cv. ‘HZ’) and a female variety of Nekken2 (*Oryza sativa* L. subsp. *japonica* cv. ‘Nekken2’) as the parents. Germination under 80 mM NaCl conditions was measured and analyzed, and quantitative trait locus (QTL) mapping was completed using a genetic map. A total of 16 salt-tolerance QTL ranges were detected at bud stage in rice, which were situated on chromosomes 3, 4, 6, 8, 9, 10, 11, and 12. The maximum limit of detection was 4.69. Moreover, the *qST12.3* was narrowed to a 192 kb region on chromosome 12 using map-based cloning strategy. Statistical analysis of the expression levels of these candidate genes under different NaCl concentrations by qRT-PCR revealed that *qST12.3* (*LOC_Os12g25200*) was significantly down-regulated with increasing NaCl concentration, and the expression level of the chlorine-transporter-encoding gene *LOC_Os12g25200* in HZ was significantly higher than that of Nekken2 under 0 mM NaCl. Sequencing analysis of *LOC_Os12g25200* promoter region indicated that the gene expression difference between parents may be due to eight base differences in the promoter region. Through QTL mining and analysis, a plurality of candidate genes related to salt tolerance in rice was obtained, and the results showed that *LOC_Os12g25200* might negatively regulate salt tolerance in rice. The results provide the basis for further screening and cultivation of salt-tolerant rice varieties and have laid the foundation for elucidating further molecular regulation mechanisms of salt tolerance in rice.

## Introduction

Soil salinization is a global problem that severely restricts the cultivation of rice and is one of the predominant abiotic stresses affecting the rice crop yield (Munns and Gilliham, 2015; Yang and Guo, 2018). According to reported statistics, about 100 million hm^2^ of the world’s land is affected by salinization. About 10 million hm^2^ of this land is in China, accounting for about 10% of global soil salinization regions, which severely limits food productivity in China (Hu et al., 2018; Liu et al., 2021; Zou et al., 2021). Rice (*Oryza sativa* L.), a salt-sensitive crop, is a gramineous plant and one of the three most essential staple crops in China and throughout the world (Tian et al., 2011; Ganie et al., 2019). It is most susceptible to salt stress at the seedling stage and heading stage (Zou et al., 2021). Salt tolerance is a complicated physiological process. When under salt stress, the rice in cellular salt content disturbs the cells’ ionic and osmotic stability, having an effect on the health and productivity of plants. At this time, plant cells try to maximize the dynamic stability of ions, and thus plant health, by inducing gene expression (Wu et al., 2013; Singh et al., 2021). Those attempting to increase the yield and quality of rice face new challenges (Rao et al., 2020), and efforts to enhance the salt tolerance of rice will assist in maximizing the use of saline-alkali land, and hence, improve crop yields (Radanielson et al., 2018; Wang et al., 2019). Therefore, exploring the mechanisms of rice salt tolerance is anticipated to provide a theoretical groundwork that will lead to a realistic way to cultivate salt-tolerant rice types, enhancing rice yield in saline land and, consequently, securing food production in China and other parts of the world.

Seed germination is critical, in which the catabolism of proteins, starches or oils accumulated by seeds during maturation supports the growth of early seedlings. Therefore, seed germination is also the basis for the total biomass and total production of plants throughout their life cycles (Bewley, 1997; Penfield, 2017). In the process of seed germination, the high salt environment will lead to the disorder of various metabolic pathways in plants, change the enzyme activities, and the leakage of high solutes, resulting in the reduction of K^+^ efflux, and hinder the activity of α-amylase (Gomes-Filho et al., 2008). Some studies identified about 50 loci have been identified for seed germination under salt stress by genome-wide association studies (GWAS) (Shi et al., 2017; Yu et al., 2018). He et al. (2019) located a QTL *qSE3* during the germination and seedling stage of rice seeds under salt stress, cloned and isolated *qSE3* and found that it encoded the K^+^ transporter *OsHAK21*. *qSE3* significantly enhanced the uptake of Na^+^ and K^+^ by germinating seeds under salt stress. Zeng et al. (2021) identified 13 QTLs from the local cultivar Wujiaozhan, and a major salt-tolerance specific QTL *qGR6.2* was precisely mapped. These studies provided information on the genetic basis of marker-assisted selection to improve rice seed germination under salt stress.

Salt tolerance in rice is a complex quantitative trait regulated by multiple genes, and the expression of these related genes is closely related to the environment (Qi et al., 2008; Tiwari et al., 2016). Salt stress can cause changes to the activity of electron transport chains in mitochondria and chloroplasts and destroy the dynamic balance between the generation and removal of reactive oxygen species (ROS), leading to protein oxidation, membrane damage, and DNA fragmentation, and other disruptions (Mishra et al., 2013). At present, QTL mapping studies have found the highest number of genes related to salt tolerance on chromosomes 2 and 6, and the fewest genes related to salt tolerance on chromosomes 10, 11, and 12 (Hu et al., 2018). Abdur et al. (Razzaque et al., 2011) found that high salt stress affected the balance of Na^+^, K^+^, and Cl^-^ in the roots of rice plants. Na^+^ was the main ion that caused harm to rice under salt stress (Munns and Tester, 2008), and Na^+^ transporters such as HKTs, NHXs and ROS scavengers play key roles in maintaining cell homeostasis under salt stress and positively affect the ability of plants to cope with increased salinity. High concentrations of Na^+^ prevent the normal functioning of rice biofilms, affect the normal absorption of nutrients, and lead to physiological metabolic disorders (Bharathkumar et al., 2016). A high concentration of Cl^-^ was shown to affect the normal growth and development of the rice plant. The influencing mechanisms for this might be anion mediation of the periodic regulation and inhibition of ribosomal enzymes that catalyze protein synthesis by cells (Geilfus, 2018). Joo et al. (2014) found that CatA, CatB, and CatC were all involved in the regulation of plant abiotic stress. Some results indicated that the Cat protein was also involved in the response of plants to salt stress (Nagamiya et al., 2007). Zhou et al. (2018) found that the receptor-like cytoplasmic kinase STRK1 phosphorylated and activated CatC which then positively regulated the salt tolerance of rice, which has a practical significance in rice yield and quality.

In this study, recombinant inbred line (RIL) populations constructed using Huazhan (HZ) and Nekken2 rice varieties as parents were used to locate and analyze QTL for salt tolerance. This was achieved by examining the response of rice seeds to salt stress at the germination stage and creating a high-density single nucleotide polymorphism (SNP)-based molecular marker map. Candidate genes were screened and expression was analyzed for QTL clusters with high effector values to ascertain the identity of additional QTL and genes related to the regulation of salt stress and provide a molecular research basis and theoretical support for the screening of salt-tolerant rice germplasm resources.

## Materials and methods

### Experimental materials

Huazhan (*Oryza sativa* L. subsp. *indica* cv. ‘HZ’) is a kind of super rice variety, which has strong adaptability, better resistance to adversity and stress than ordinary varieties, and better resistance to diseases and pests. It belongs to the varieties with high and stable yield. Nekken2 (*Oryza sativa* L. subsp. *japonica* cv. ‘Nekken2’) is a kind of rice with strong affinity and strong spectral affinity, and its F_1_ seed setting rate is up to 80%, which has important utilization value in Chinese rice breeding of super high yield (Sun et al., 1992). In this experiment, an HZ plant was used as the male parent to hybridize with Nekken2, used as the female parent, and obtain the F_1_ generation. The F_1_ generation was bagged and selfed *via* the single-seed descent method, and 120 stably inherited recombinant inbred lines were obtained after continuous selfing for 12 generations to form the RIL population (Wang et al., 2020).

### Rice cultivation and management

For each strain of rice seed, 60 seeds were taken from each of the 120 strains of F_12_ and both parents, rinsed 2-3 times with 70% alcohol, and surface-disinfected with 10% sodium hypochlorite solution. The seeds were then rinsed well with deionized water. After 1 month, 24 seedlings of each strain were selected and transplanted in the experimental field, with each strain planted in four rows of six plants. Routine water and fertilizer management and insect pest and weed control strategies were carried out during this period.

### Salt stress tolerant treatments

Sixty full-seeded seeds of HZ, Nekken2, and each hybrid strain were selected, hulled, disinfected with 70% alcohol for 2 minutes, disinfected with 30% sodium hypochlorite solution for 20-30 minutes, rinsed with distilled water, inoculated onto 1/2 MS medium containing 80 mM NaCl (Zhang, 2012), and incubated in an artificial climate incubator for 10 days. The germination rate of each strain was determined (germination standard: Refer to GB 5520-85 for the germination standard of rice seeds: the young roots should reach the seed length, and the young buds should reach at least half the length of the grain) (Meng et al., 2022). Three replicates were set up for each group. The incubator conditions were light/dark (14 h/10 h), 30°C, and 70% humidity.

### Genetic mapping construction

DNA from the biparental HZ and Nekken2 and 120 recombinant self-inbred line populations were extracted and the genomes re-sequenced. The genome sequencing results were sorted to obtain 4858 molecular markers evenly distributed throughout 12 chromosomes for the construction of the genetic map (Wang et al., 2020).

### QTL localization

Using a high-density SNP molecular marker linkage map method already established in the laboratory, interval mapping was used to analyze the QTL for seed germination rate separately using Mapmaker/QTL1.1B software. An LOD setting of 2.5 was applied as the threshold value to determine the presence of the QTL and we following the rules proposed by Mccouch et al. (1997) to name the QTL.

### The development of CSSLs and fine mapping for *qST12.3*

To obtain Chromosome Segment Substitution Lines (CSSL) for *qST12.3*, the plants carrying HZ genotype at the flanking region of *qST12.3* were selected to backcross with Nekken2 for 3 rounds. The SSR markers RM101 and RM1337 were simultaneously used to identify the plants containing HZ genotype in backcross lines. A set of 124 SSR markers (**Supplementary Table S1**) uniformly distributed on a previous linkage map were used to select the individual plants containing the least HZ DNA in BC_4_F_1_ lines. It contained a small amount of HZ DNA in its genetic background, and carried a homozygous introgression across the entire *qST12.3* region and without any introgression across *qST3*, *qST4.1*, *qST4.2*, *qST6*, *qST8*, *qST9*, *qST10.1*, *qST10.2*, *qST11.1*, *qST11.2*, *qST11.3*, *qST11.4*, and *qST11.5* regions on chromosomes 3, 4, 6, 6, 9, 10, and 11 respectively. Then, a total of 186 selected plants from 770 BC_4_F_2_ progenies were cultivated in the paddies to gain enough seeds for the salt stress tolerant evaluation and further fine mapping.

### Candidate gene expression analysis

Using the Total Plant RNA Extraction Kit (Axygen), total RNA was extracted from HZ and Nekken2 leaves, and 1 μg of total RNA was aspirated to reverse transcribe into cDNA using the cDNA Reverse Transcription Kit (Toyobo). Candidate genes were predicted based on the QTL localization results combined with the Rice Genome Annotation Database (http://rice.plantbiology.msu.edu/). In qRT-PCR, the SYBR Green Realtime PCR Master Mix (Toyobo) and the primers (**Supplementary Table S2**) were used to detect the expression levels of candidate salt-tolerance-related genes, with rice *OsActin* as the internal reference gene.

The total volume of the qRT-PCR reaction system was 10 μL, including 2 μL of cDNA template, 5 μL of SYBR qPCR Mix (Toyobo), 1 μL each of upstream and downstream primer (10 μmol/L^-1^), and 1 μL of ddH_2_O.

The qRT-PCR reaction program was 95°C for 30 s, 95°C for 5 s, 55°C for 10 s, 72°C for 15 s, and 45 cycles. Three replicates were set up for each reaction and relative quantification was performed using the 2^-ΔΔCT^ method (Livak and Schmittgen, 2001). The data obtained were analyzed for significant differences using Excel for t-tests and GraphPad Prism 6 software for graphical analysis. *P*<0.05 indicates a significant difference, while *P*<0.01 indicates a highly significant difference.

### Salt gradient stress treatment

Sixty seeds of each parent, HZ and Nekken2, were sterilized and inoculated onto 1/2 MS medium containing 0 mM, 60 mM, 80 mM, or 100 mM NaCl for 14 days. Candidate genes were analyzed for expression. Based on difference in the expression of candidate genes between the parents, candidate genomic DNA fragments were amplified from both parents using high-fidelity DNA polymerase KOD-Plus-Ver.2 (Toyobo) and the PCR products were sequenced.

## Results and analysis

### Phenotypes of both parents and RIL groups

**Figure 1:**
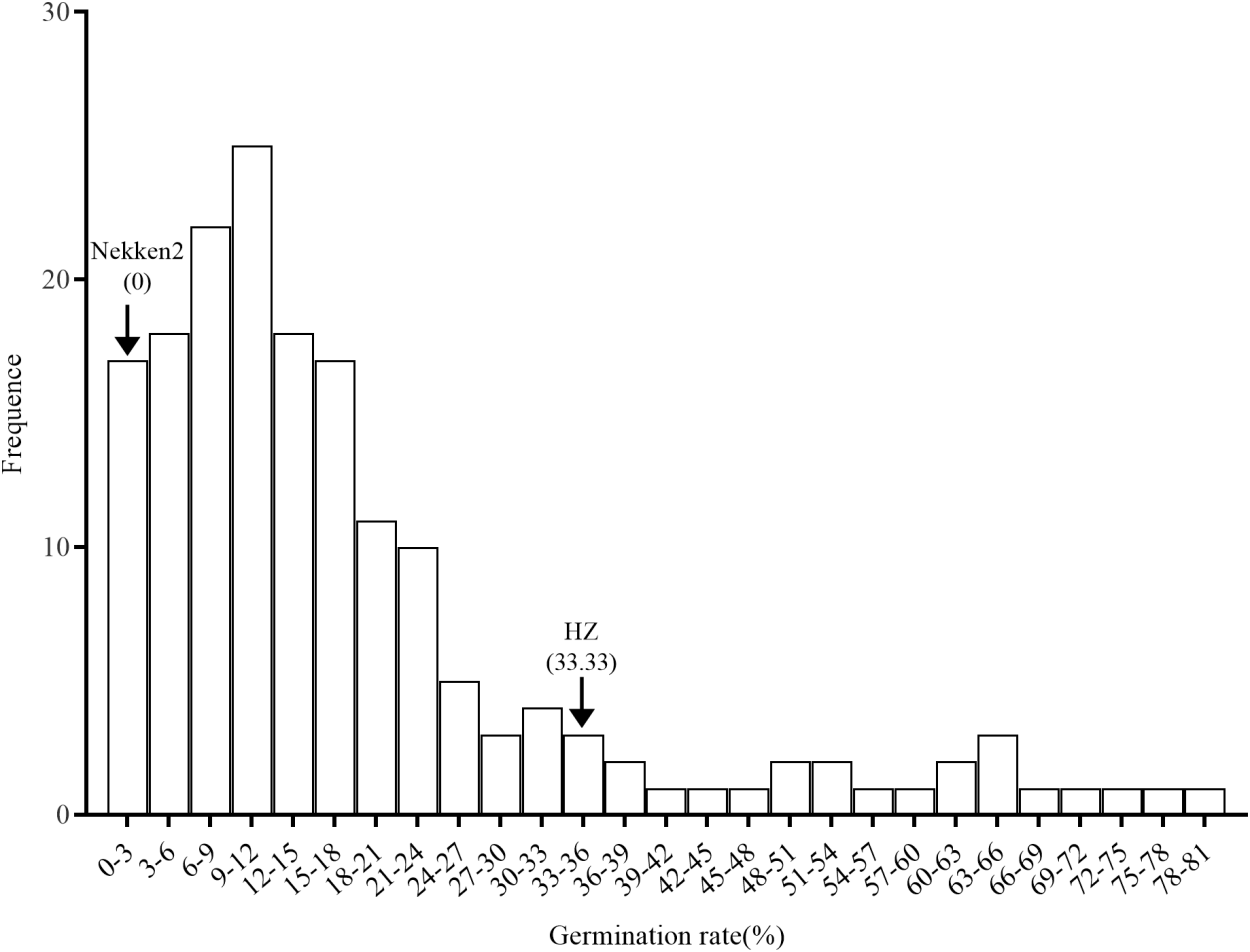
Germination rate distribution of rice recombinant inbred lines under salt treatment.

**Figure 2:**
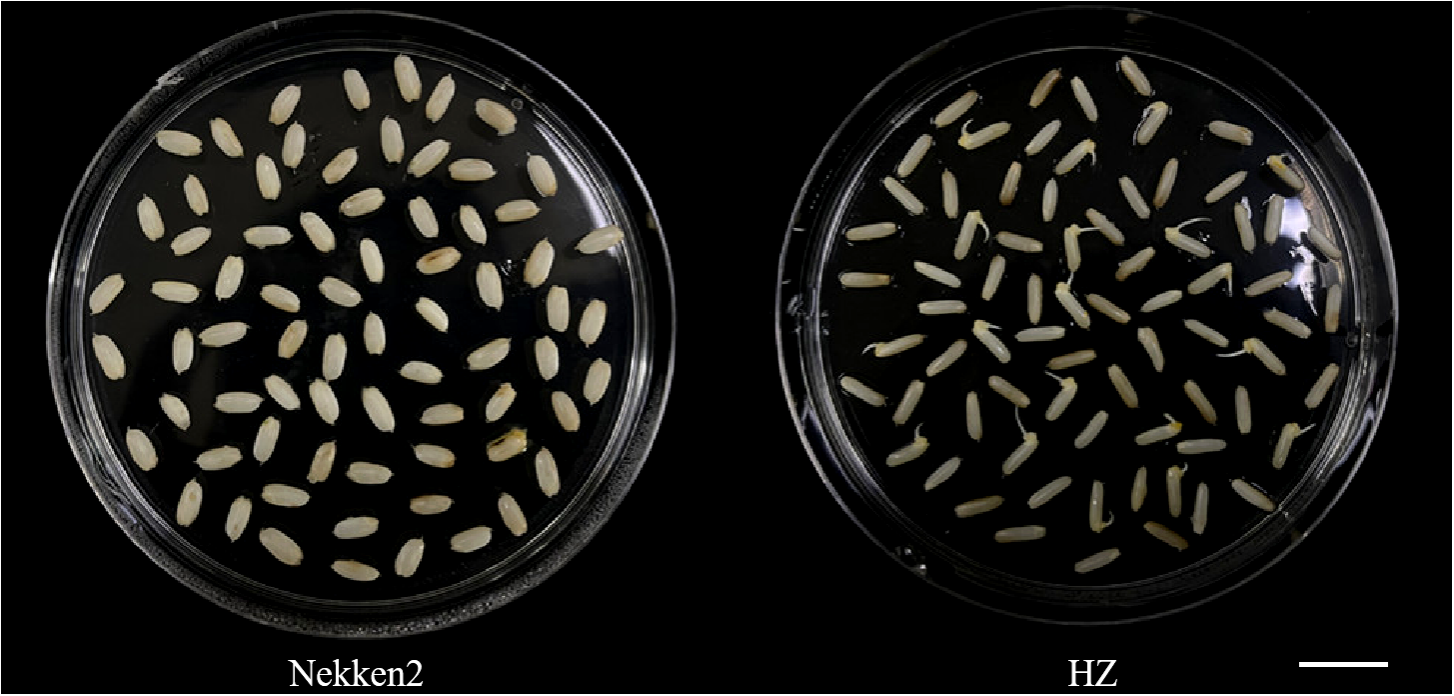
Phenotype diagram of germination rate of Huazan and Nekken2 under 80mM salt stress, bar=5cm.

The results of salt-tolerant treatments of both parents showed that there was a significant difference between HZ and Nekken2 (**Figure 1**). The germination rates (GR) of seeds after salt treatment were found to be 0% for Nekken2 and approximately 33.33% for HZ, the latter of which was presumed to be more salt tolerant than Nekken2 at a concentration of 80 mM NaCl (**Figures 2**, **S1**). The germination rate data for each strain of the RIL population under salt stress showed a continuous normal distribution, and several strains showed super parental germination rates. In addition, the genetic characteristics of the quantitative traits were consistent with the requirements of QTL interval mapping.

### Salt tolerance QTL localization analysis

**Figure 3:**
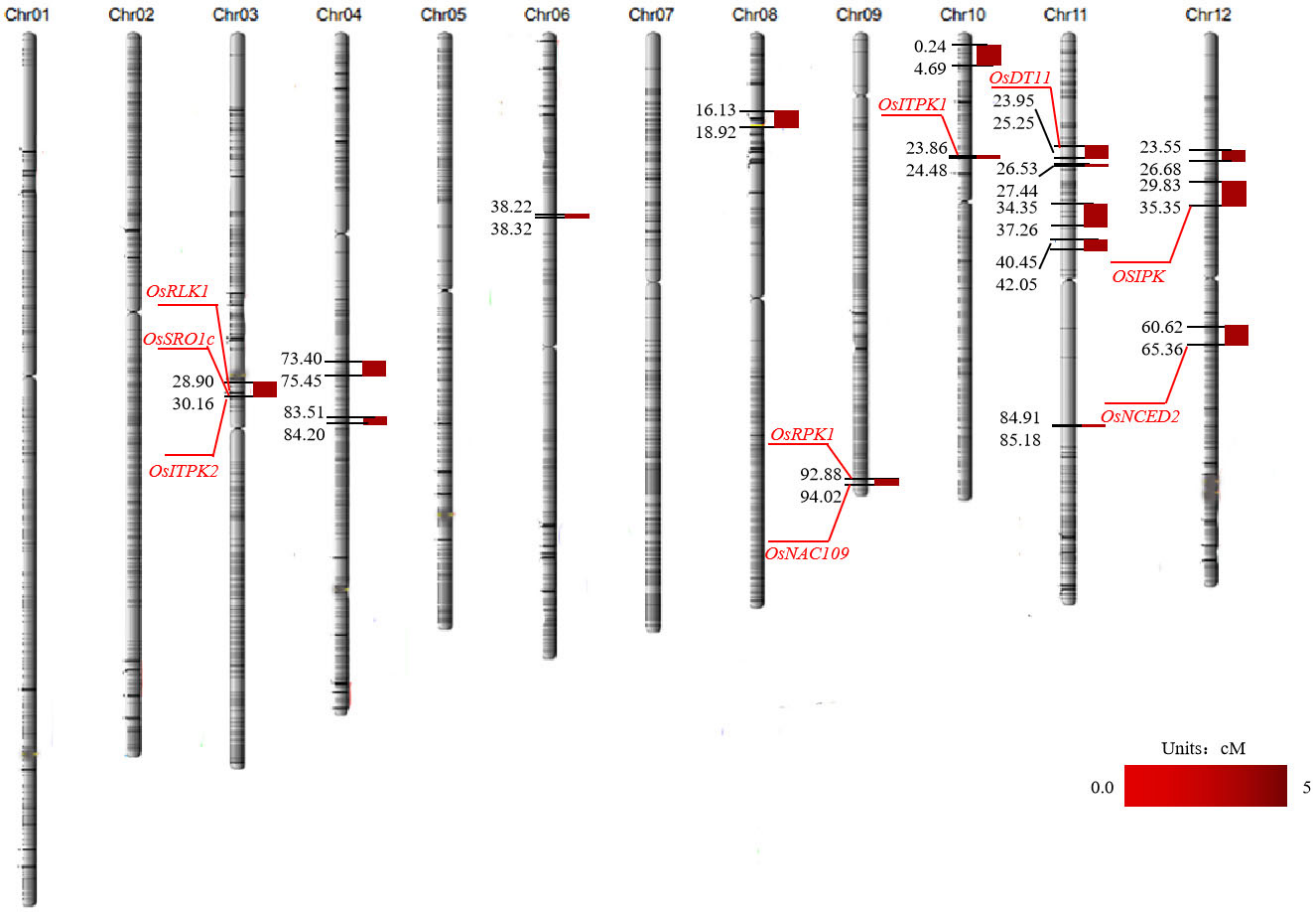
Mapping of QTL for salt tolerance in rice. The red gene number is the cloned salt tolerance gene screened in the salt tolerance interval.

A total of 16 QTLs related to salt tolerance were detected on chromosomes 3, 4, 6, 8, 9, 10, 11, and 12 using the molecular linkage map established in our laboratory. The highest LOD value was 4.69 and was located with the genetic distances 23.86 cM and 24.48 cM on chromosome 10 (**Table 1**; **Figure 3**).

**Table 1:** Mapping of QTL for salt tolerance in rice.

QTL	Chr	Physical distance (bp)	Position of support (cM)	LOD
*qST3*	3	6743718~7034710	28.90~30.16	3.69
*qST4.1*	4	17123204~17600552	73.40~75.45	3.32
*qST4.2*	4	19482788~19642144	83.51~84.20	3.16
*qST6*	6	8917905~8938061	38.22~38.32	2.61
*qST8*	8	3764126~4411594	16.13~18.92	2.94
*qST9*	9	21667334~21932045	92.88~94.02	2.80
*qST10.1*	10	57733~1093124	0.24~4.69	3.05
*qST10.2*	10	5567413~5709027	23.86~24.48	4.69
*qST11.1*	11	5588569~5889092	23.95~25.25	2.59
*qST11.2*	11	6190775~6314547	26.53~27.44	2.82
*qST11.3*	11	8013951~8692604	34.35~37.26	2.67
*qST11.4*	11	9438113~9808325	40.45~42.05	2.82
*qST11.5*	11	19809355~19870739	84.91~85.18	3.16
*qST12.1*	12	5494931~6224960	23.55~26.68	2.59
*qST12.2*	12	6959601~8245853	29.83~35.35	2.83
*qST12.3*	12	14141879~15247966	60.62~65.36	3.00

### Functional analysis of candidate genes

Based on the results of the QTL localization analysis and fine mapping, the location of the salt tolerance interval was determined, and the functions of the genes in each QTL interval were analyzed in conjunction with the rice gene database (http://rice.plantbiology.msu.edu/). The functions of and physiological information on these genes were briefly summarized (**Table 2**). Eight of these genes were cloned, including *LOC_Os03g12730* (*OsRLK1*), *LOC_Os03g12820* (*OsSRO1c*), and *LOC_Os03g12840* (*OsITPK2*) on chromosome 3, *LOC_Os09g37949* (*OsRPK1*) and *LOC_Os09g38000* (*OsNAC109*) on chromosome 9; *LOC_Os10g01480* (*OsITPK1*) on chromosome 10, *LOC_Os11g10590* (*OsDT11*) on chromosome 11, *LOC_Os12g12860* (*OSIPK*) and *LOC_Os12g24800* (*OsNCED2*) on chromosome 12 (**Table 2**; **Figure 3**). Moreover, the *qST12.3* was fine-mapped to a 192 kb region on rice chromosome 12 using map-based cloning strategy.

**Table 2:** Functional analysis of candidate genes in QTL mapping interval.

Gene ID	Chr	Function	Cloned or not
*LOC_Os03g12730*	3	Leucine-rich repeat receptor-like protein kinases	Have been cloned
*LOC_Os03g12820*	3	(SRO) SRO protein; SNAC1 target gene; Callus Browning	Have been cloned
*LOC_Os03g12840*	3	1,3,4-phosphoinositol 5/6-kinase gene	Have been cloned
*LOC_Os09g37949*	9	Receptor protein kinase	Have been cloned
*LOC_Os09g38000*	9	NAC transcription factor	Have been cloned
*LOC_Os10g01480*	10	1,3,4-phosphoinositol 5/6-kinase gene	Have been cloned
*LOC_Os10g01760*	10	Peroxidase precursors	Not cloning
*LOC_Os11g10590*	11	Drought tolerance	Have been cloned
*LOC_Os11g10640*	11	A protein containing a protein kinase domain	Not cloning
*LOC_Os12g10660*	12	B-box zinc finger protein	Not cloning
*LOC_Os12g10740*	12	Leucine-rich repeating family of proteins	Not cloning
*LOC_Os12g12860*	12	Calcium-dependent protein kinase	Have been cloned
*LOC_Os12g13570*	12	MYB family transcription factors	Not cloning
*LOC_Os12g24800*	12	9-cis-epoxy carotenoid dioxygenase gene *OsNCED2*	Have been cloned
*LOC_Os12g25200*	12	Chlorine transporters, a family of chlorine channels	Not cloning

### Candidate gene expression level analysis

**Figure 4:**
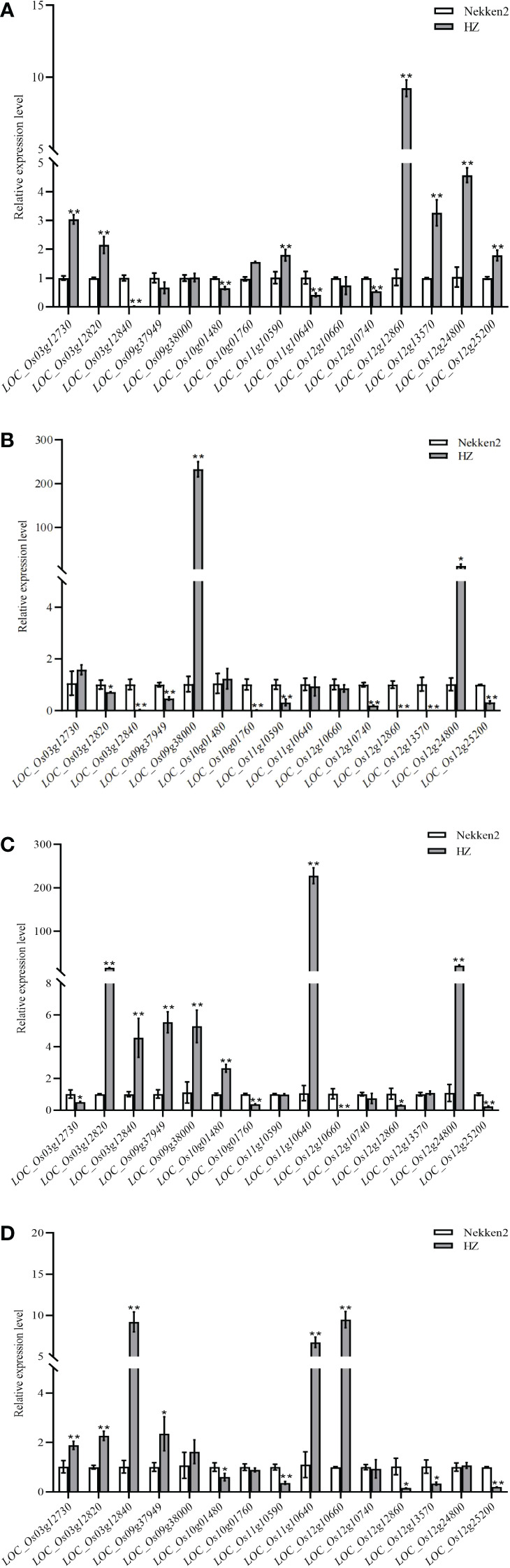
Expression of candidate genes for salt tolerance in rice under different concentrations of NaCl. **(A)** Expression of candidate genes at 0 mM NaCl concentration. **(B)** Expression of candidate genes at 60 mM NaCl concentration. **(C)** Expression of candidate genes at 80 mM NaCl concentration. **(D)** Expression of candidate genes at 100 mM NaCl concentration. * indicates significance at *P* ≤ 0.05 by Student’s *t* test, ** indicates significance at *P* ≤ 0.01 by Student’s *t* test.

Analysis of salt tolerance candidate genes by qRT-PCR after 14 days of treatment with 0, 60, 80 or 100 mM salt stress of both parents, HZ and Nekken2, revealed (**Figures 4A–D**) that, at 0 mM NaCl, *LOC_Os03g12730*, *LOC_Os03g12820*, *LOC_Os03g12840*, *LOC_Os10g01480*, *LOC_Os11g10590*, *LOC_Os11g10640*, *LOC_Os12g10740*, *LOC_Os12g12860*, *LOC_Os12g13570*, *LOC_Os12g24800*, and *LOC_Os12g25200* were significantly differentially expressed in the two parents; at 60 mM NaCl, *LOC_Os03g12820* and *LOC_Os12g24800* were significantly differentially expressed in the two parents, and *LOC_Os03g12840*, *LOC_Os09g37949*, *LOC_Os09g38000*, *LOC_Os10g01760*, *LOC_Os11g10590*, *LOC_Os12g10740*, *LOC_Os12g12860*, and *LOC_Os12g13570*, *LOC_Os12g25200* were very significantly differently expressed in the two parents; at 80 mM NaCl concentration, *LOC_Os03g12730*, *LOC_Os12g12860* had significantly different in expression in the two parents, and *LOC_Os03g12820*, *LOC_Os03g12840*, *LOC_Os09g37949*, *LOC_Os09g38000*, *LOC_Os10g01480*, *LOC_Os10g01760*, *LOC_Os11g10640*, *LOC_Os12g10660*, *LOC_Os12g24800*, and *LOC_ Os12g25200* were very significantly different in expression in the two parents; and at 100 mM NaCl concentration, *LOC_Os09g37949*, *LOC_Os10g01480*, *LOC_Os12g12860*, and *LOC_Os12g13570* were expressed significantly differently between the two parents, and *LOC_Os03g12730*, *LOC _Os03g12820*, *LOC_Os03g12840*, *LOC_Os11g10590*, *LOC_Os11g10640*, *LOC_Os12g10660*, *LOC_Os12g25200* were significantly differentially between the two parents.

Under 0 mM NaCl, the expression of *LOC_Os12g25200* in HZ was significantly higher than that in Nekken2. After treatment with 60, 80, or 100 mM NaCl, the expression of this gene decreased significantly in HZ. The expression of this gene decreased sequentially with the increase in salt concentration and reached the lowest expression at 100 mM NaCl. The quantitative results showed that, with increasing salt concentration, the resistance of the plants to salt stress was maintained by a decrease in *LOC_Os12g25200* expression in HZ, which maintained the normal growth and development of the plants.

### Sequence analysis of the candidate gene *LOC_Os12g25200*

**Figure 5:**
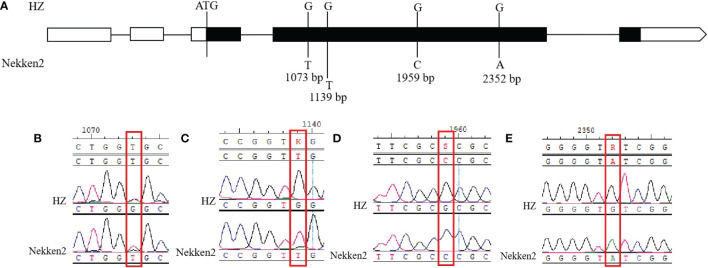
Sequence analysis of *LOC_Os12g25200* gene. The black region is the exon region, where single base mutations occur. **(A)** DNA loci difference analysis of *LOC_Os12g25200* gene. **(B)** Site difference at 1073 bp. **(C)** Site difference at 1139 bp. **(D)** Site difference at 1959 bp. **(E)** Site difference at 2352 bp.

Analysis of the effects of stress treatment with the different concentrations of salt *via* quantitative qRT-PCR showed that the expression of *LOC_Os12g25200* was significantly higher in HZ than in Nekken2 in the absence of salt stress, while expression of this gene gradually decreased with increasing salt concentration, suggesting this may be a novel gene that negatively regulates salt tolerance in rice. Therefore, the *LOC_Os12g25200* gene in HZ and Nekken2 was sequenced and analyzed for locus differences, and a total of four single-base differences were found in the exons (**Figures 5A–E**), located at 1073 bp (G in HZ vs T in Nekken2), 1139 bp (G in HZ vs T in Nekken2), 1959 bp (G in HZ va C in Nekken2), and at 2352 bp (G in HZ vs A in Nekken2). Among these, the single-base differences at 1073 bp and 1139 bp caused amino acid changes, located at position 60 (glycine in HZ vs valine in Nekken2) and position 82 (tryptophan in HZ vs leucine in Nekken2), respectively, while in the protein, the other two single-base differences did not cause amino acid changes. Therefore, it was speculated that the difference in the expression of the *LOC_Os12g25200* gene in HZ and Nekken2 may be caused by differences in the amino acid sequences encoded by their genes, which in turn leads to differences in salt tolerance between HZ and Nekken2. In order to further explore the reasons for the difference in the expression level of this gene between parents, we sequenced the first 2200 bp promoter sequence of *LOC_Os12g25200* initiation codon and a total of eight single-base differences were found in the exons (**Supplementary Figures S2A–H**), located at 733 bp (C in HZ vs A in Nekken2), 937 bp (A in HZ vs T in Nekken2), 1005-1006 bp (Nekken2 is deficiency G and A), 1058 bp (C in HZ vs T in Nekken2), 1119 bp (G in HZ vs A in Nekken2), 1174 bp (C in HZ vs T in Nekken2), 1605 bp (C in HZ vs T in Nekken2) and at 1979 bp (A in HZ vs G in Nekken2). We speculated that the single base differences in the eight promoter regions above might lead to the differences in gene expression levels between parents.

### Substitution mapping of *qST12.3* and analyzing candidate genes

**Figure 6:**
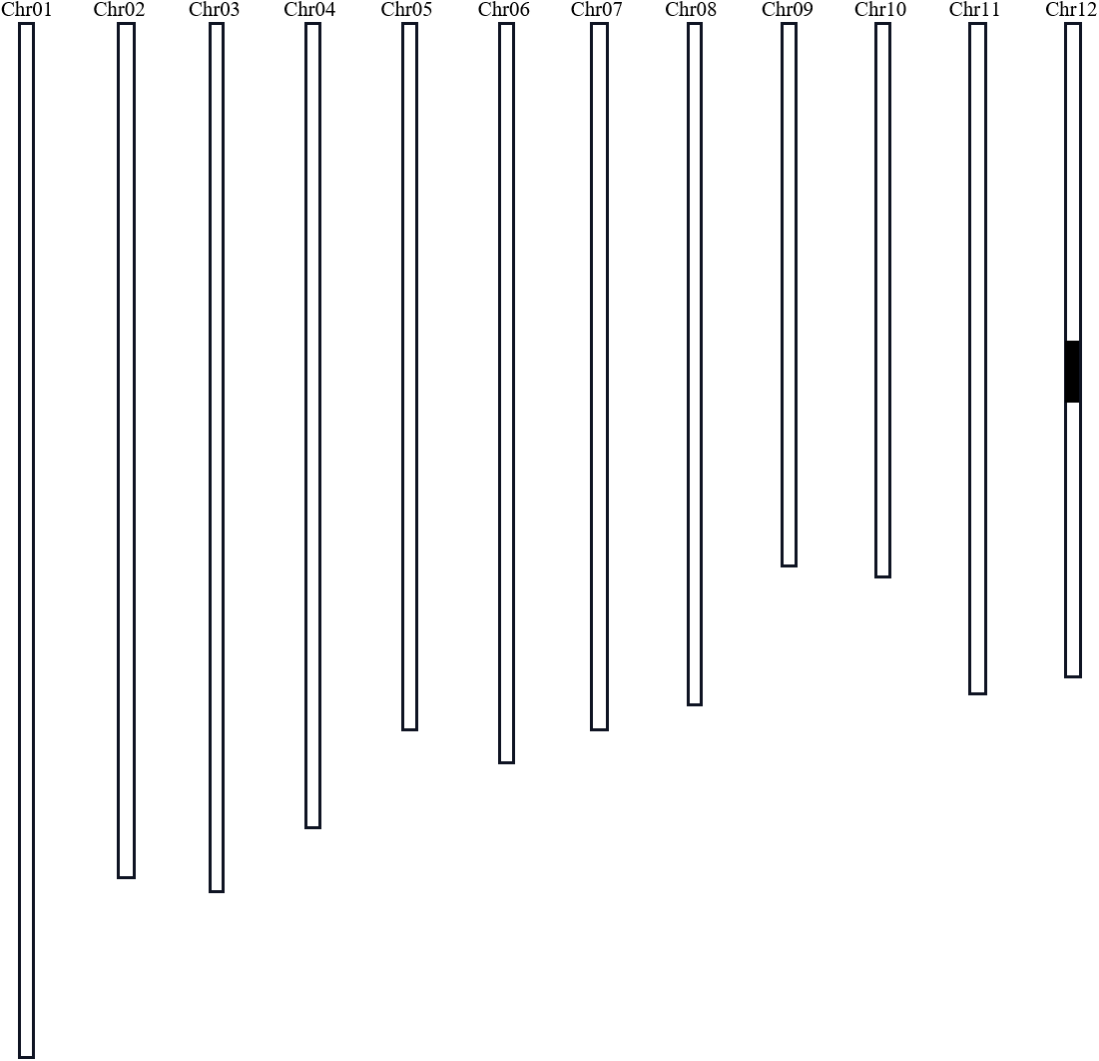
Graphical genotype of CSSL12 (a substitution line of chromosome 12). Black bar indicates the genome fragment from HZ; the other parts were from Nekken2.

**Figure 7:**
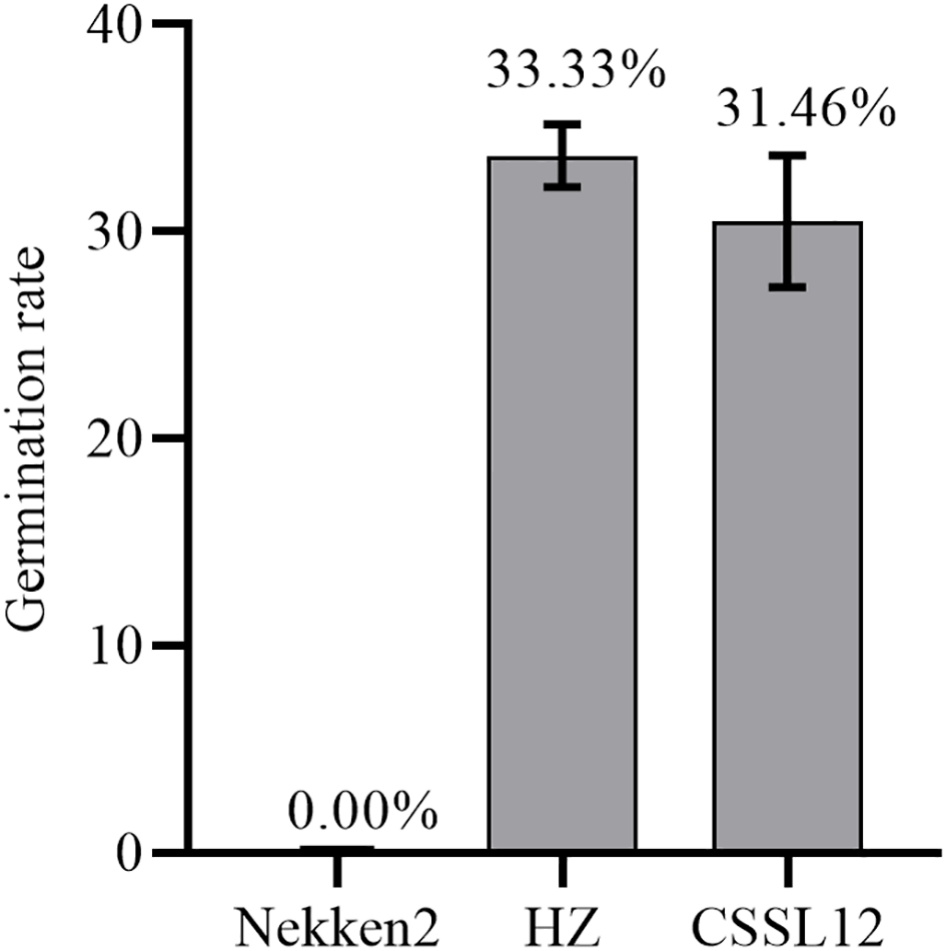
The germination rate for Nekken2, HZ and CSSL12.

Three lines from the RIL population were selected to backcross with Nekken2 for 4 rounds. According to the QTL analysis and a previous genetic linkage map, the SSR markers RM101 and RM1337 were used in marker-assisted selection for segregating the progenies carrying *qST12.3* during every generation of backcross. After 4 rounds of backcrosses with Nekken2, the BC_4_F_1_ and BC_4_F_2_ generations were scanned with a set of 124 SSR markers, which were uniformly distributed on a previous linkage map (**Supplementary Table S1**). The plant CSSL12 containing a small amount of HZ DNA was selected. It carried a homozygous introgression across the entire *qST12.3* region, but without any introgression across *qST3*, *qST4.1*, *qST4.2*, *qST6*, *qST8*, *qST9*, *qST10.1*, *qST10.2*, *qST11.1*, *qST11.2*, *qST11.3*, *qST11.4*, and *qST11.5* regions on chromosomes 3, 4, 6, 8, 9, 10, and 11, respectively (**Figure 6**). The GR of CSSL12 was significantly higher than that of its recurrent parent Nekken2, but was much more similar to that of HZ (**Figure 7**).

**Figure 8:**
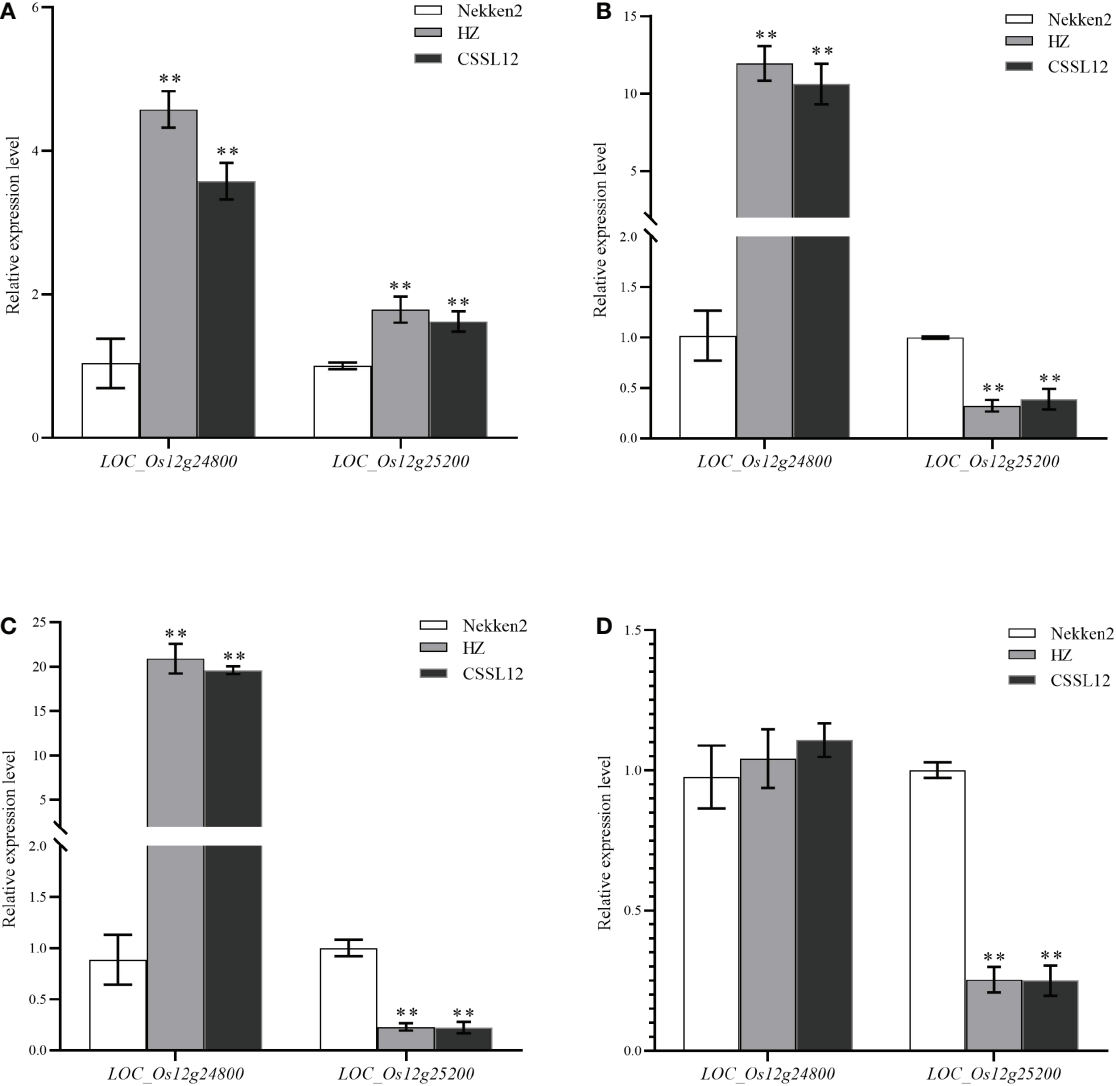
The qRT-PCR analysis of predicted genes in Nekken2, HZ and CSSL12. **(A)** Expression of candidate genes at 0 mM NaCl concentration. **(B)** Expression of candidate genes at 60 mM NaCl concentration. **(C)** Expression of candidate genes at 80 mM NaCl concentration. **(D)** Expression of candidate genes at 100 mM NaCl concentration. * indicates significance at *P* ≤ 0.05 by Student’s *t* test, ** indicates significance at *P* ≤ 0.01 by Student’s *t* test.

The target region contains 2 predicted genes (*LOC_Os12g24800* and *LOC_Os12g25200*) based on the Rice Genome Annotation Website (http://rice.plantbiology.msu.edu/). The *LOC_Os12g24800* (*OsNCED2*) has been demonstrated by Sandra et al. (Oliver et al., 2007) to increase the ABA level in anthers of Doongara rice after low temperature treatment, which proved that *LOC_Os12g24800* can regulate endogenous ABA in plants under cold stress. We performed a quantitative reverse transcription-PCR (qRT-PCR) analysis, the results show that *LOC_Os12g24800* and *LOC_Os12g25200* indicated significantly different expression levels in HZ and CSSL12 compared with Nekken2 (**Figures 8A–D**). The expression levels for *LOC_Os12g25200* decreased with the increase of salt stress concentration. In conclusion, *LOC_Os12g25200* is likely to be the candidate genes for *qST12.3.*

## Discussion

Salt tolerance in rice is a quantitative trait regulated by multiple genes. After treating HZ and Nekken2 with different concentrations of salt, analysis using qRT-PCR revealed significant differences in the expression of candidate genes in the two parents. Different treatments, such as the different growing environments of rice, differences between parents, and the determination of the germination rate at each salt concentration could affect the results of the QTL localization to varying degrees. In this experiment, we used salt stress tolerance as the only indicator for QTL detection. Based on the results of QTL interval localization, we combined with qRT-PCR and mapping of substitution lines, we explored and analyzed possible genes involved in regulating salt stress tolerance in rice to provide a theoretical basis for expanding the use of land with high soil salinity and improving the yield and quality of rice under this condition.

In this study, QTL localization was performed using a population of RILs composed of HZ and Nekken2 hybrids based on germination rate statistics under salt treatment conditions, resulting in 16 QTL intervals associated with salt tolerance, of which six QTL loci overlapped with those described by previous studies. Singh et al. (2021) located QTL intervals associated with salt tolerance at the seedling and reproductive stages of rice by meta-QTL, *mQTL4.7* on chromosome 4 at 83.2 cM, *mQTL11.2* on chromosome 11 at 40.96 cM, and *mQTL12.3* on chromosome 12 at 60.06 cM are similar to the loci on chromosomes 4, 11, and 12 found in this study. Gimhani et al. (2016) showed *mQTL12.3* which was associated with the salinity survival index (SSI) and percent damaged shoots (PDS) under salt stress, was located at the uppermost 0.63 cM region of chromosome 10, and this partially overlaps with *qST4.2*, *qST11.4*, and *qST12.3* which were respectively localized to chromosomes 4, 11, and 12 in this study. The loci *qSSI10* and *qPDS10* associated with the SSI and PDS respectively, partially overlap with *qST10.1* on chromosome 10 in this study. Ziyan Xie (2020) used the BC_2_F_7_ backcross introgression population constructed from MingChuan 63 (salt-tolerant, recurrent parent) and 02428 (salt-sensitive) to characterize chromosome 12 *qSHL12.2*, *qSHW12.4*, and *qRW12.2* and metabolite-related *qM16-12.1*, *qM27-12*, *qM28-12*, *qM39-12*, *qM47-12.1*, *qM57-12*, *qM58-12.1*, *qM61-12*, *qM72-12*, *qM76-12.1*, *qM79-12.1*, *qM85-12*, and *qM87-12* intervals. Of these, *qM47-12.1* and *qM58-12.1* contributed 43.0% and 34.7%, respectively, and these partially overlap with the *qST12.2* interval localized on chromosome 12 in this study. Tiwari et al. (2016) used a population of RILs composed of CSR11/M148 to identify a salt tolerance gene encoding a rice MYB transcription factor within the *qSSIGY8.1* interval localized to chromosome 8, which is adjacent to the *qST8* interval localized to chromosome 8 in the present study.

Based on the previous studies, the present study has yielded several new loci for salt tolerance, including *qST3*, which is localized in the interval 28.90–30.16 cM on chromosome 3; *qST4.1* in the interval 73.40–75.45 cM on chromosome 4; *qST9* in the interval 92.88–94.02 cM on chromosome 9; *qST10.2*, in the interval 23.86–24.48 cM on chromosome 10, with an LOD value of 4.69, indicating the likely presence of salt tolerance-related master genes; *qST11.1*, *qST11.2*, *qST11.3*, and *qST11.5* on chromosome 11; and *qST12.1* on chromosome 12 in the interval of 23.55–26.68 cM.

Analysis in conjunction with the Rice Gene Database (http://rice.plantbiology.msu.edu/) revealed that *LOC_Os12g25200*, a functional gene contained on QTL interval *qST12.3* encoding a chloride transporter protein of the chloride channel protein family overlaps with the interval locus reported by Singh et al. (Tian et al., 2011) The *LOC_Os12g25200* gene, which may be involved in osmotically regulating the transport of anionic chloride ions in rice and is associated with the regulation of salt tolerance in rice. Combined with qRT-PCR analysis of the expression of this gene in HZ and Nekken2 under different salt stress conditions, our results suggested that *LOC_Os12g25200* showed significant differences in expression between the two parents, and the expression of *LOC_Os12g25200* in HZ was significantly higher than that in Nekken2 under 0 mM NaCl stress. After treatment with 60, 80, or 100 mM NaCl stress gradients, the expression of *LOC_Os12g25200* decreased significantly in HZ and declined sequentially with increasing salt stress concentrations, reaching the lowest expression at 100 mM NaCl. Golldack et al. (2003) compared transcriptional changes in the salt-sensitive rice strain IR29 and the salt-tolerant strain Pokkali and identified the expression of *OsCLC1* in rice as a means to investigate Cl^-^ homeostasis under salt stress conditions. Analysis of the transcripts revealed that anion channels may also be involved in the uptake of anions by cells. The above findings indicate that *OsCLC1* plays an important role in the coordinated regulation of anion and cation homeostasis in rice under abiotic salt stress, suggesting an osmoregulatory function for *OsCLC1* in high salt stress environments. This has some similarities with the function of the protein encoded by *LOC_Os12g25200* found in our study, suggesting that *LOC_Os12g25200* may be involved in a similar mechanism in HZ, reduction in the expression of this gene improved the salt tolerance of rice. The above findings need to be further verified by subsequent relevant experiments.

To further verify the molecular mechanisms of *LOC_Os12g25200* in improving salt stress tolerance in rice, we sequenced the gene and analyzed changes in the gene sequence. Sequencing *LOC_Os12g25200* revealed four single-base differences between HZ and Nekken2 at exons 1073 bp, 1139 bp, 1959 bp, and 2352 bp, respectively, with “G to T” changes occurred at 1073 bp and 1139 bp, respectively. The amino acids encoded at positions 60 and 82 both showed “glycine to valine” changes, while the remaining two single-base differences did not cause amino acid changes. Meanwhile, we sequenced the promoter region of this gene and found that there were 8 base differences between HZ and Nekken2 at 733 bp, 937 bp, 1005–1006 bp, 1058 bp, 1119 bp, 1174 bp, 1605 bp and 1979 bp within the first 2200 bp of the start codon. It is speculated that these differences may lead to the difference in the expression of this gene in parents. The above sequencing results indicate that single-base differences the DNA sequences and promoter sequences of the *LOC_Os12g25200* candidate gene indeed lead to changes in the encoded amino acids, and these differences may be important to differences in gene expression and thus the salt stress tolerance in HZ and Nekken2. Previous studies have indicated that Yin et al. (2022) measured the QTL interval localization related to root traits without NaCl treatment, and the QTL interval that they mapped did not overlap with the one in this study. These results indicated that the QTL interval mapped in this study was caused by the treatment with 80 mM NaCl, and further ruled out the difference in interval location caused by the genotype. This also laid the foundation for the subsequent cloning of salt tolerance genes.

In this study, *qST12.1*, *qST12.2* and *qST12.3* related to salinity tolerance at three seed bud stages were reported, which a potential *qST12.3* controlled GR. The QTL on chromosome 12 partially overlaps with results from previous studies (Livak and Schmittgen, 2001). Based on the QTL interval mapped by RIL population, candidate genes were screened out for real-time fluorescence quantitative analysis of their expression changes under salt stress. Furthermore, the CSSL12 substitution line was constructed based on *qST12.3*, and the results showed that it was a salt tolerance QTL controlling seed germination on chromosome 12 of rice. At the same time, we found that *LOC_Os12g25200* was a novel gene, and the expression of CSSL12 was different between the parents and the substitution line according to the gene transcription level. A candidate gene *LOC_Os12g25200* for a chloride transporter in a family of chloride channels was identified. Therefore, additional tests are needed to test whether *LOC_Os12g25200* is a major gene for *qST12.3* tolerance in seed germination.

The reaction mechanism of rice plants to salt stress is complex. Osmotic stress is one of the main stresses that plants face in the early stage of salt stress (Ahmed et al., 2015). Although some QTLs have been applied to high-yield rice varieties to improve salt tolerance, salt tolerance in rice is a complex trait controlled by multiple genes and affected by environmental factors. At present, there is no explanation for this complex regulatory mechanism, and improvement progress is slow. Therefore, the identification and cloning of major genes related to salt tolerance in rice will help to explain the unknown mechanism of salt tolerance and apply it to breeding.

## Conclusion

Using a population of RILs composed of HZ and Nekken2 hybrids in this study, several new salt tolerance loci were located, among which a gene encoding a chloride transporter protein, with chloride channel family function, was found in the *qST12.3* interval, a locus that overlapsd with those described in previous studies. It was found that the expression of *LOC_Os12g25200* decreased significantly with increasing salt concentration and was less experessed in Nekken2 than HZ, by constructing the CSSL12 substitution line population, we further proved that there was a major gene controlling salt tolerance in the major effect interval of *qST12.3*, suggesting that this gene may play an important role in negatively regulating salt stress tolerance in rice and may be the main reason for the difference in salt stress tolerance between the two strains. The results of this experiment further uncovered QTL loci related to salt tolerance and confirmed the functions of genes related to salt stress tolerance in rice, the study provides a new theoretical basis and has application value for elucidating the mechanism of salt stress tolerance in rice. The results of this research may be applicable to practical rice molecular breeding and could provide key genetic loci that can be used to improve the ability of rice to tolerate salt stress. This can ultimately greatly icrease the land area that can be used for food production, greatly enhance the yield and quality of rice, and has important practical significance for the screening and breeding of excellent rice varieties.

## Data availability statement

The datasets presented in this study can be found in online repositories. The names of the repository/repositories and accession number(s) can be found in the article/**Supplementary Material**.

## Author contributions

WY, TiL, ZC, TaL, and HY conceived and designed the experiment. YM, YL performed data collection. WY, ML, and XZ produced data analysis and figures. WY and TiL wrote the original manuscript with input from XY, YR and YW. All authors contributed to the article and approved the submitted version.
