# Supplementary material for: Quantitative trait locus mapping and candidate gene analysis for salt tolerance at bud stage in rice
Source: Front Plant Sci. 2023 Jan 16;13:1041081. doi: 10.3389/fpls.2022.1041081 (PMC9886062; doi:10.3389/fpls.2022.1041081)
Supplement: Supplementary file 1 [file DataSheet_1.pdf]

ADDITIONAL FILE FIGURE TABLE

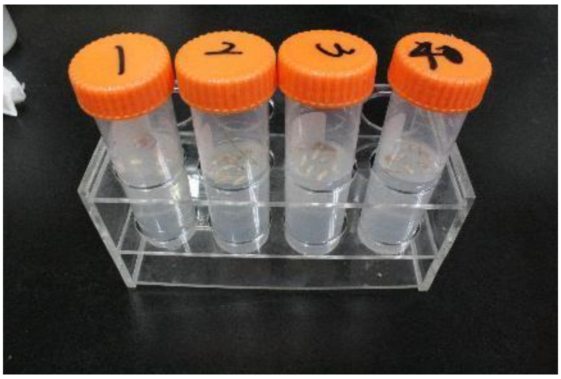

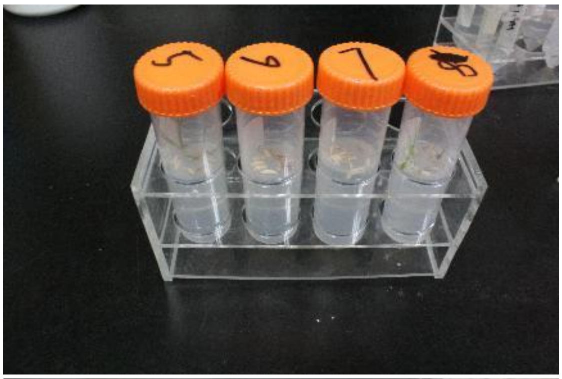

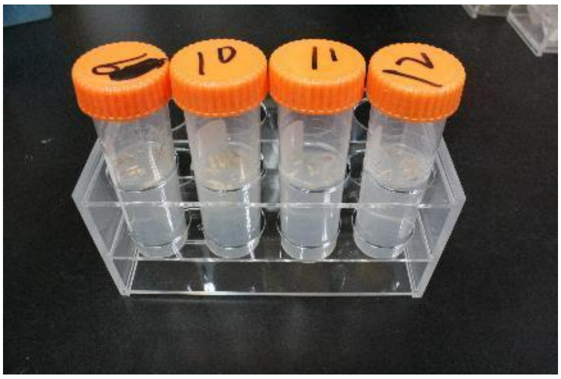

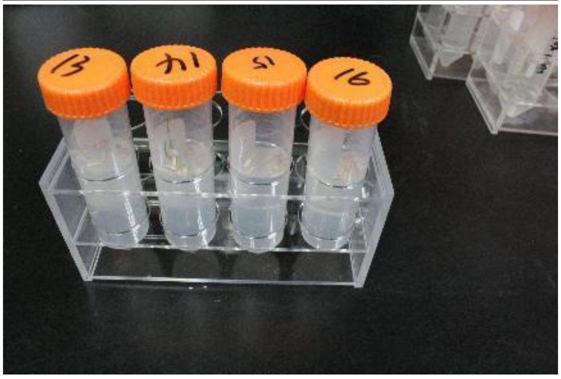

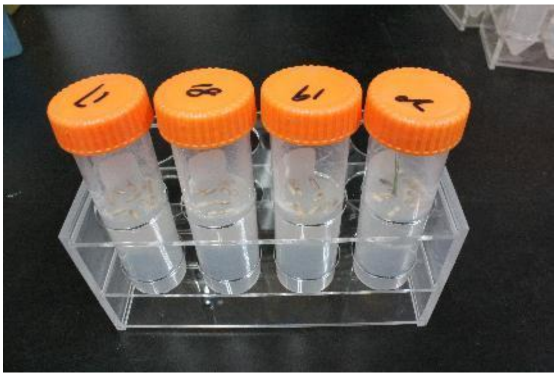

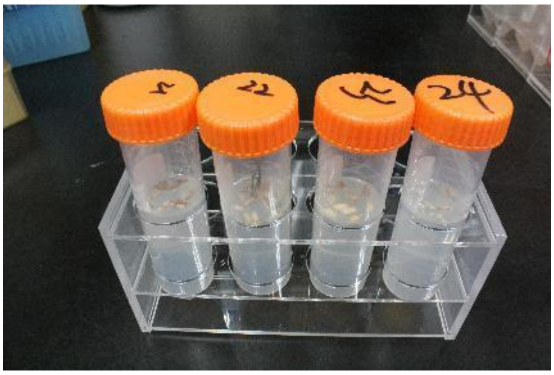

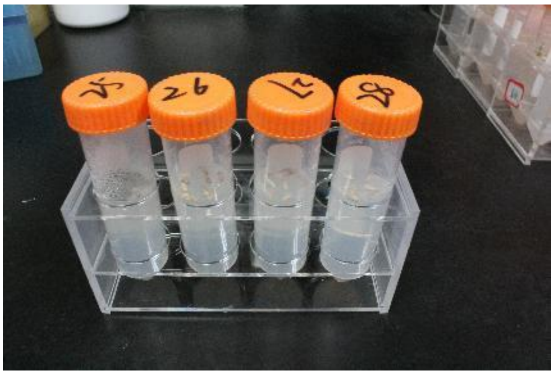

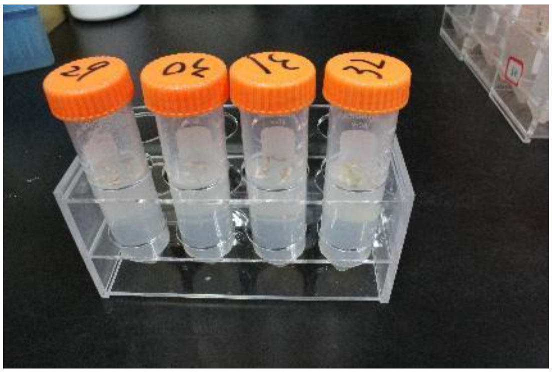

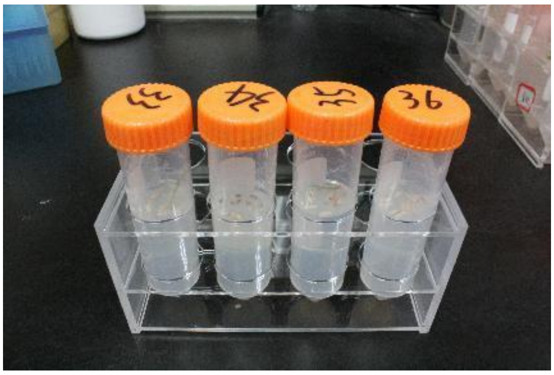

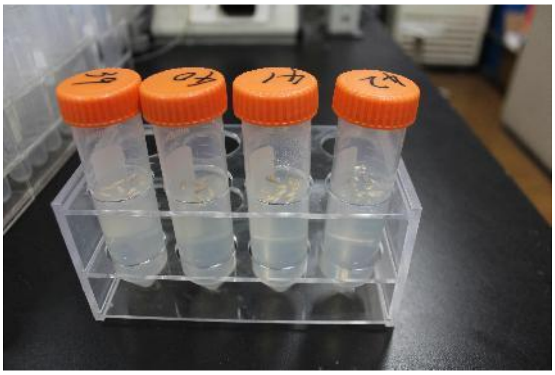

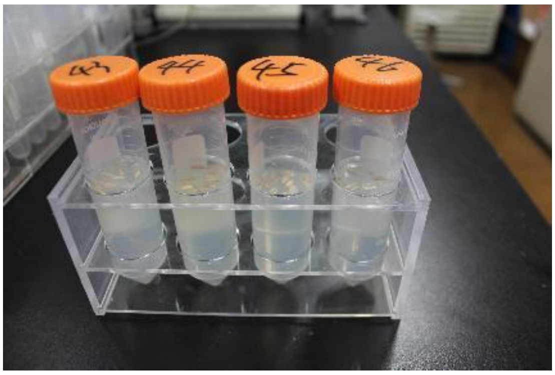
43
44
45
46
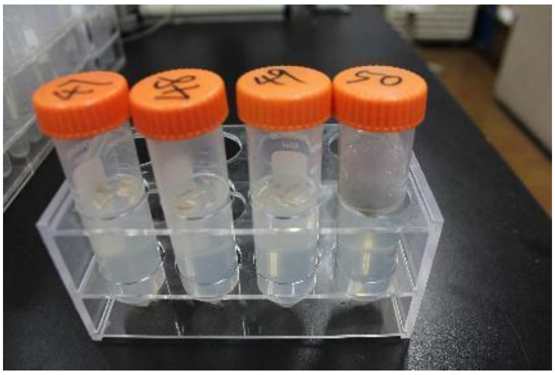
49
50
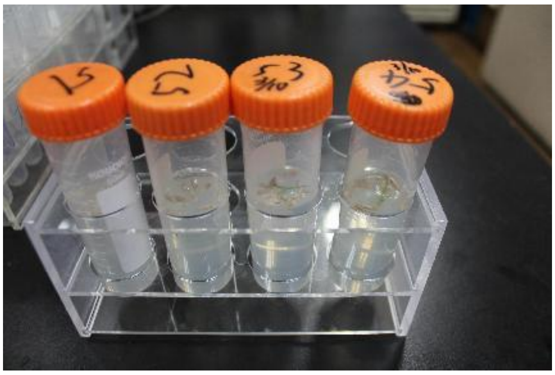
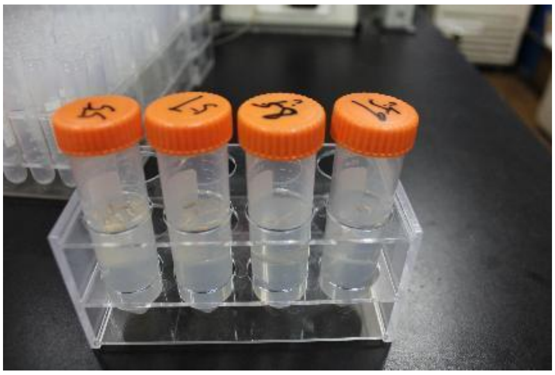
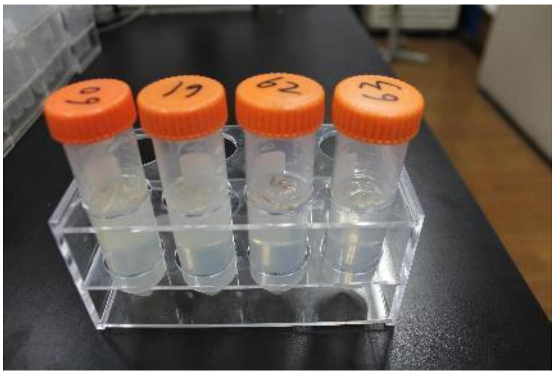
62
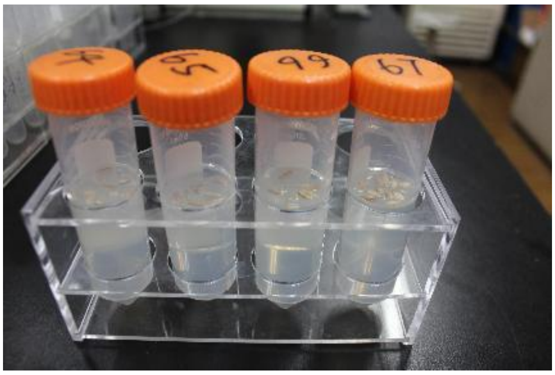
67
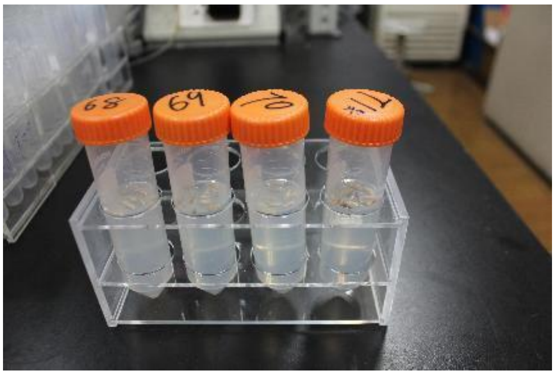
68
69
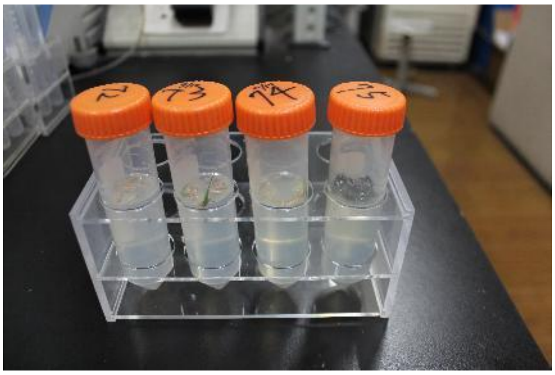
73
74
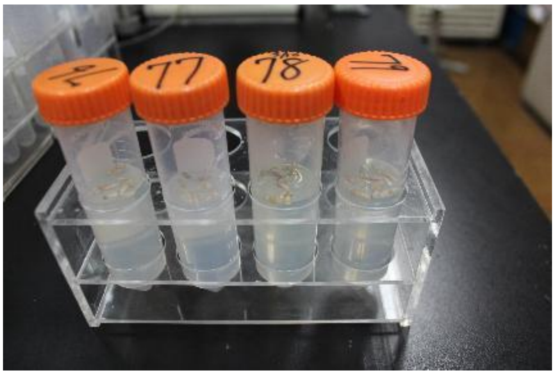
77
78
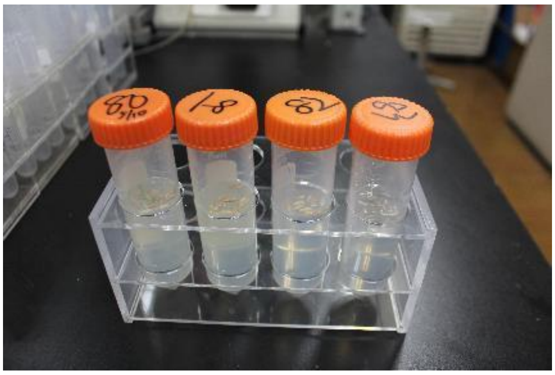
80

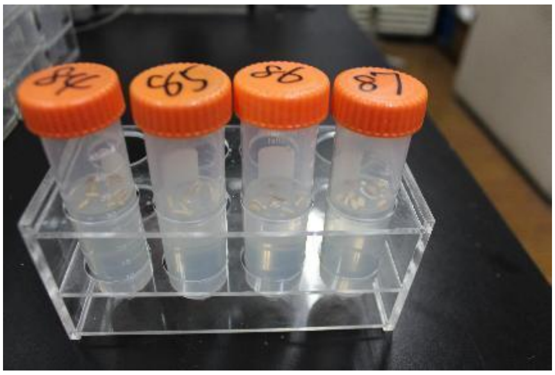

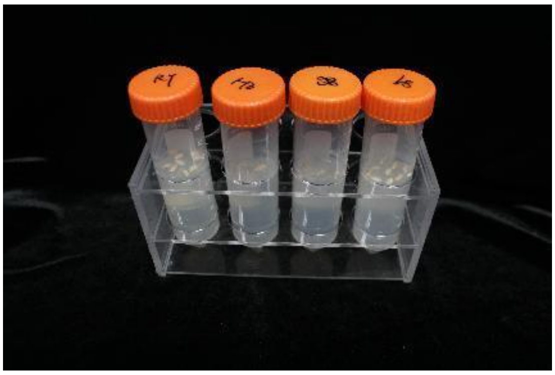

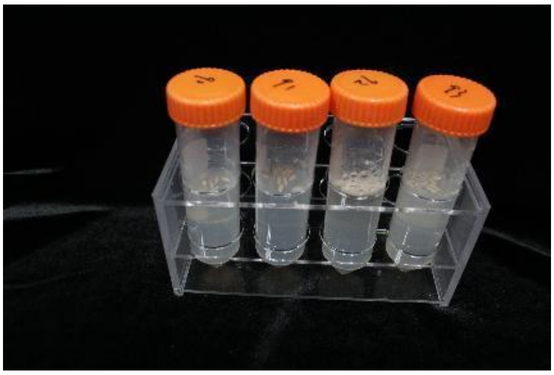

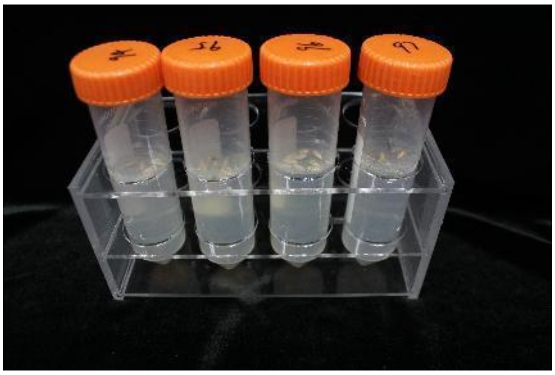

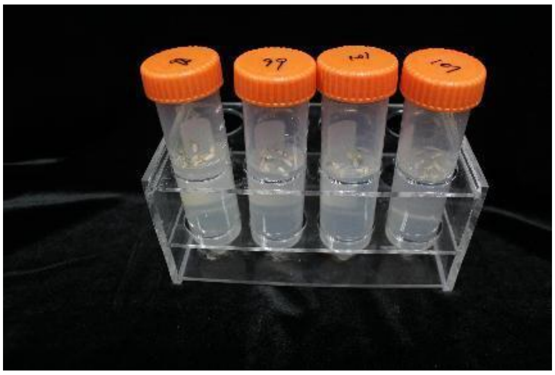

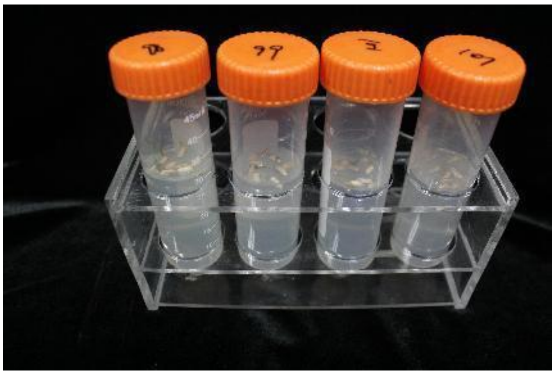

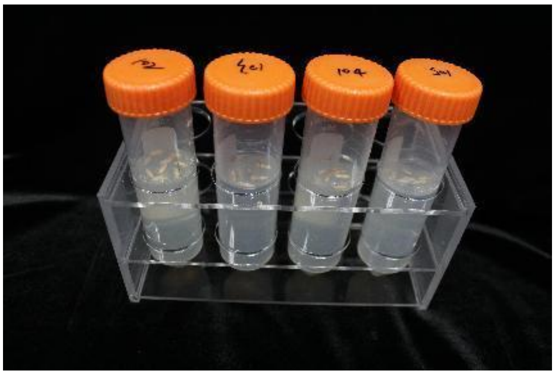

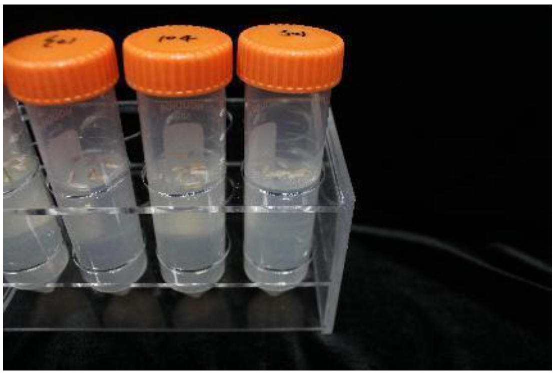

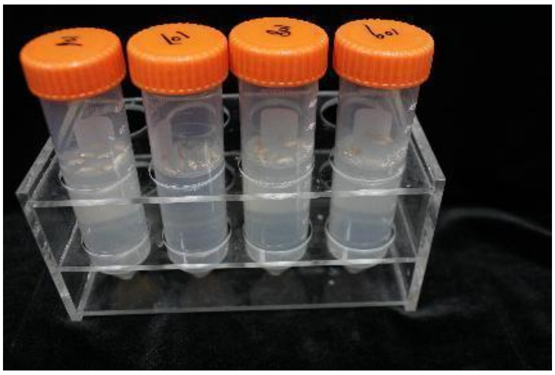

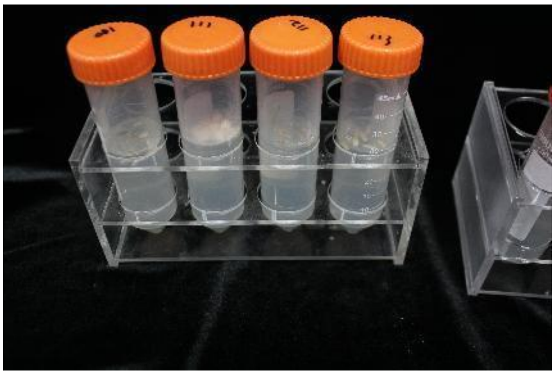

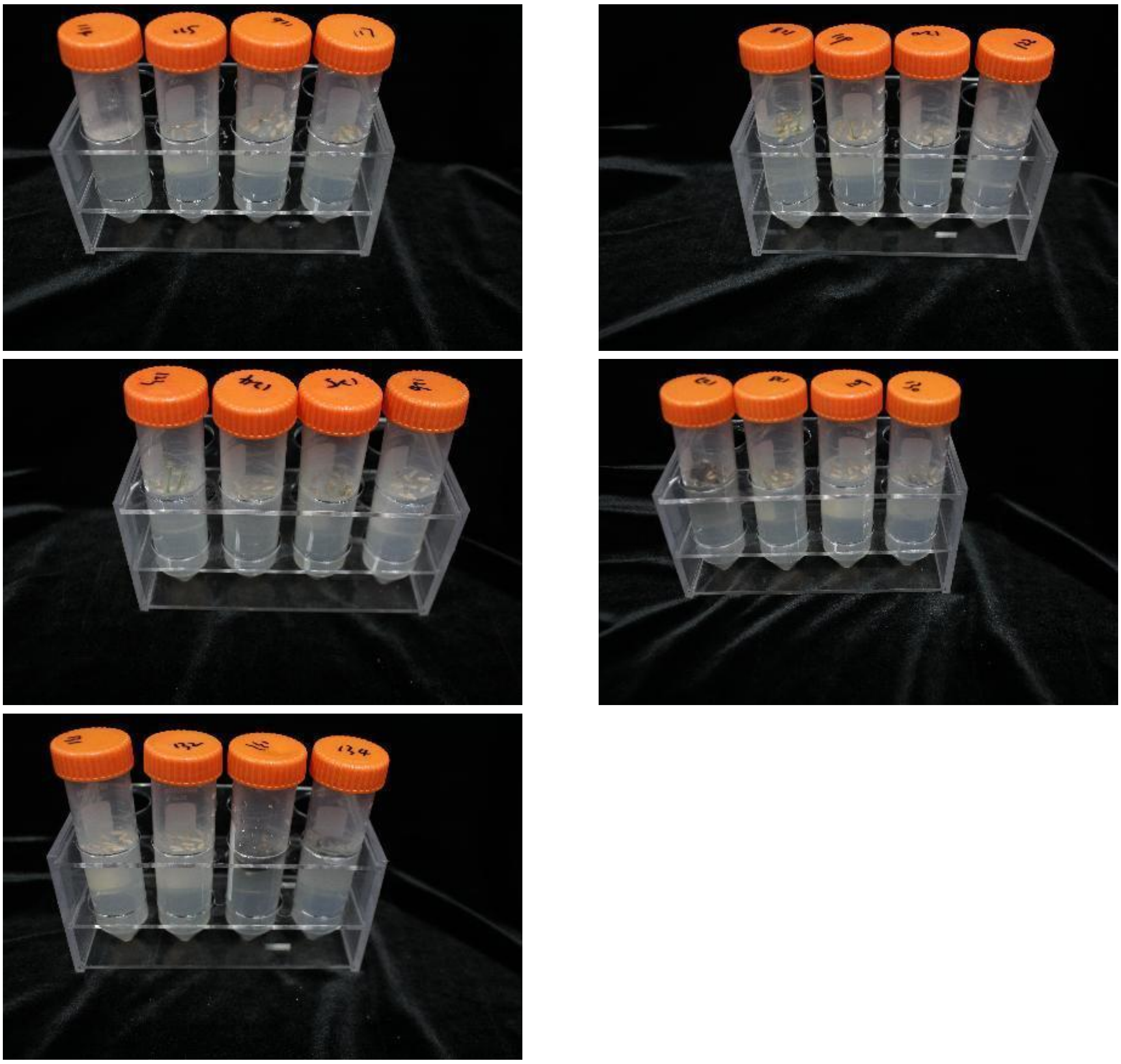

Figure S1. Phenotypes of both parents and RIL populations treated with 80mM NaCl.

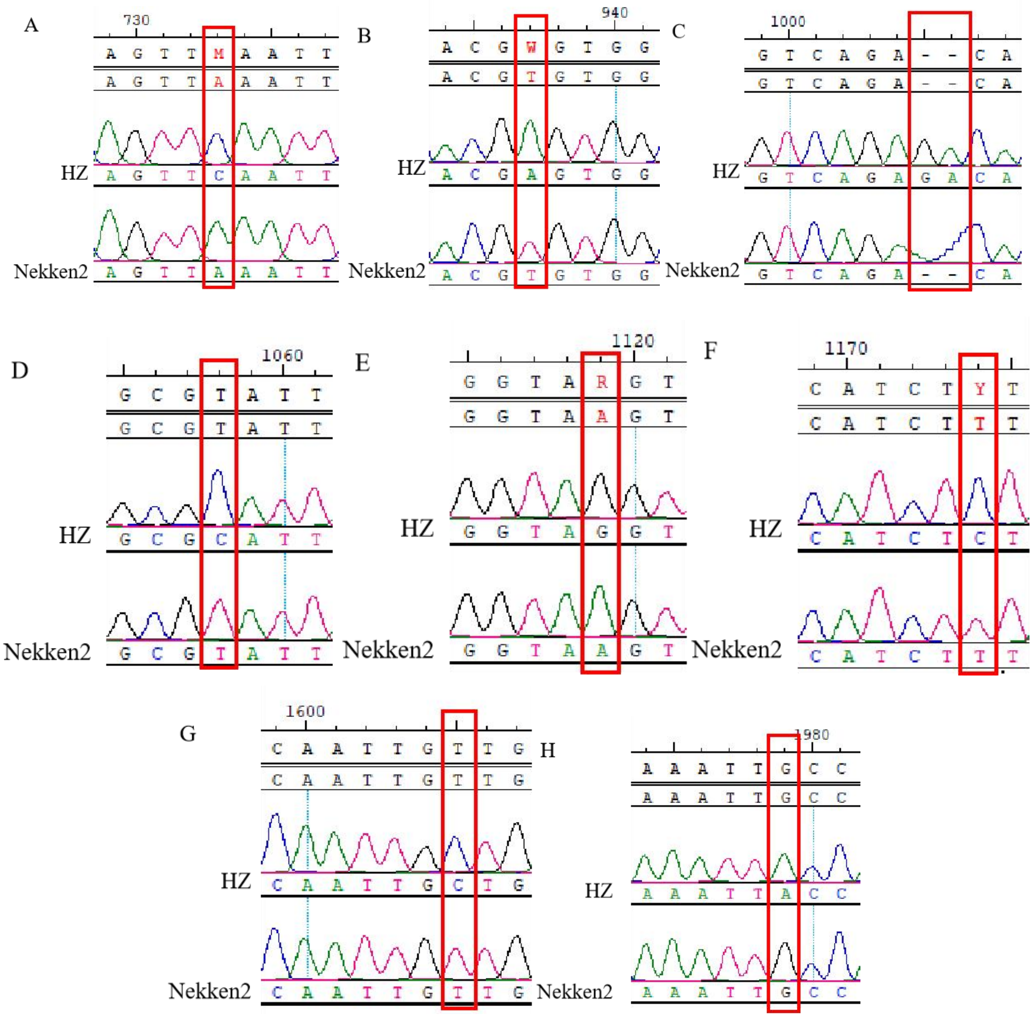

Figure S2. Sequencing of *LOC_Os12g25200* promoter region.

Table S1. SSR markers selected to identify the CSSLs.

Chromosome	SSR markers
Chr.01	RM3252, RM1247, RM3740, RM6902, RM8111, RM6786, RM583, RM580, RM259, RM493, RM6880, RM8097, RM6703, RM3738, RM1361, RM8100, RM6321
Chr.02	RM7581, RM300, RM262, RM3515, RM6318, RM1920, RM3763, RM6933, RM3316
Chr.03	RM14795, RM6676, RM3513, RM1350, RM3856, RM5172
Chr.04	RM8213, RM307, RM16874, RM1155, RM6997, RM3524, RM3839, RM303, RM317, RM349,RM1113
Chr.05	RM17713, RM17798, RM13, RM17954, RM18384, RM3437, RM430, RM3695, RM18751, RM3476, RM6972, RM3616, RM3809, RM334, RM19221
Chr.06	RM19234, RM585, RM217, RM276, RM19715, RM2523, RM3183, RM3496, RM162, RM5371, RM6395, RM3343, RM412
Chr.07	RM5490, RM5055, RM5711, RM6574, RM3755, RM6835, RM11, RM3826, RM1132, RM234, RM3555, RM1306
Chr.08	RM1019, RM6356, RM3819, RM5556, RM7285, RM5808, RM556, RM80, RM3571
Chr.09	RM444, RM219, RM1328, RM6051, RM3700, RM242, RM201, RM3744
Chr.10	RM6370, RM2125, RM4455, RM5689, RM1375, RM1873, RM6737, RM3451, RM4771
Chr.11	RM286, RM167, RM4504, RM6272, RM6094
Chr.12	RM5795, RM247, RM101, RM1337, RM1986, RM7376, RM1103, RM5479, RM3739, RM1226

Table S2. Primers used in the study.

QTL	Gene ID	Forward primer (5'→3')	Downstream primer (5'→3')
	OsActin	TGGCATCTCAGCACATTCC	TGCACAATGGATGGGTCAGA
qST3	*LOC_Os03g12820*	CCGTCGACAACCTTGAGAAT	AGGGAACATGTCGCGAGTAG
qST3	*LOC_Os03g12840*	ACGAGGAGGAAGTGGTGATG	ATCAAATGGGCCTTGTTCTG
qST9	*LOC_Os09g37949*	TCCACGAGATTGAGTTGCTG	ACACTGATCGGCAGATTTCC
qST9	*LOC_Os09g38000*	TCCGGAGTGGTATTTCTTCG	GACCAGCGTCTTCTTCATCC
qST10.2	*LOC_Os10g01480*	ACTAACGTTGCCCTTGGTTG	TGCCATCATTGTTTTCCAGA
qST10.2	*LOC_Os10g01760*	TTCCTCCACCTCGTCTCTTC	GGCACGTCTCGTCGTAGAAT
qST11.1	*LOC_Os11g10590*	CACTAAGATGGCGGTGGTG	GGACGAAACATGCAAAAACC
qST11.1	*LOC_Os11g10640*	TGGAACATCACCTGTTTGGA	TGTATGAATCGTGCGGGTTA
qST12.1	*LOC_Os12g10660*	GTCATCGTCAGCCCAACAAG	GCTGGAGGAGTGAGGTTCAG
qST12.1	*LOC_Os12g10740*	CCTTGAGCAAGCTCCAAAAC	GGAAGTCCTCCAGTGAGCTG
qST12.2	*LOC_Os12g12860*	CTGGGAGAAGCAGTCAGGTC	AAGTGCCCCTTCTTTTTGGT
qST12.2	*LOC_Os12g13570*	AGTGCTGCCAAAAGCTTCAT	TCGCTCAATACGCAAAACTG
qST12.3	*LOC_Os12g24800*	ATGGAAACGAGGATAGTGGT	CTTATTGTTGTGCGAGAAGT
qST12.3	*LOC_Os12g25200*	AGGAGATGGGGATGTACGTG	ATCTCGGGTCCTTGGAACTT
